# Molecular characterization of *Sarcocystis* species from Polish roe deer based on *ssu rRNA* and *cox1* sequence analysis

**DOI:** 10.1007/s00436-014-3966-x

**Published:** 2014-06-20

**Authors:** Rafał Kolenda, Maciej Ugorski, Michał Bednarski

**Affiliations:** 1 Faculty of Natural Sciences, Brandenburg University of Technology Cottbus—Senftenberg, Großenhainer Str. 57, 01968 Senftenberg, Germany; 2 Department of Biochemistry, Pharmacology and Toxicology, Wrocław University of Environmental and Life Sciences, 50-375 Wrocław, Poland; 3 Laboratory of Glycobiology and Cell Interactions, Ludwik Hirszfeld Institute of Immunology and Experimental Therapy, Polish Academy of Sciences, 53-114 Wrocław, Poland; 4 Department of Epizootiology and Clinic of Bird and Exotic Animals, Wrocław University of Environmental and Life Sciences, 50-375 Wrocław, Poland

**Keywords:** *Sarcocystis*, *ssu rRNA*, *cox1*

## Abstract

Sarcocysts from four Polish roe deer were collected and examined by light microscopy, small subunit ribosomal RNA (*ssu rRNA*), and the subunit I of cytochrome oxidase (*cox1*) sequence analysis. This resulted in identification of *Sarcocystis gracilis*, *Sarcocystis oviformis*, and *Sarcocystis silva*. However, we were unable to detect *Sarcocystis capreolicanis*, the fourth *Sarcocystis* species found previously in Norwegian roe deer. Polish sarcocysts isolated from various tissues differed in terms of their shape and size and were larger than the respective Norwegian isolates. Analysis of *ssu rRNA* gene revealed the lack of differences between *Sarcocystis* isolates belonging to one species and a very low degree of genetic diversity between Polish and Norwegian sarcocysts, ranging from 0.1 % for *Sarcocystis gracilis* and *Sarcocystis oviformis* to 0.44 % for *Sarcocystis silva*. Contrary to the results of the *ssu rRNA* analysis, small intraspecies differences in *cox1* sequences were found among Polish *Sarcocystis gracilis* and *Sarcocystis silva* isolates. The comparison of Polish and Norwegian *cox1* sequences representing the same *Sarcocystis* species revealed similar degree of sequence identity, namely 99.72 % for *Sarcocystis gracilis*, 98.76 % for *Sarcocystis silva*, and 99.85 % for *Sarcocystis oviformis*. Phylogenetic reconstruction and genetic population analyses showed an unexpected high degree of identity between Polish and Norwegian isolates. Moreover, *cox1* gene sequences turned out to be more accurate than *ssu rRNA* when used to reveal phylogenetic relationships among closely related species. The results of our study revealed that the same *Sarcocystis* species isolated from the same hosts living in different geographic regions show a very high level of genetic similarity.

**Electronic supplementary material:**

The online version of this article (doi:10.1007/s00436-014-3966-x) contains supplementary material, which is available to authorized users.

## Introduction

*Sarcocystis* spp. are obligatory intracellular protozoa, which parasitize many different animals, including mammals, birds, and fish (Tenter 1995; Fayer 2004). They have an obligatory two-host life cycle. The parasites reproduce sexually in the intestine of the carnivorous definitive host, and the asexual phase of life cycle takes place in the muscles or nervous tissue of the herbivore or omnivore intermediate hosts (Dubey et al. 1988). Pathogenic species of *Sarcocystis* cause acute disease only in intermediate hosts. Most pathogenic species of *Sarcocystis* are those infecting ruminants; in the case of massive infections, they cause hemorrhagic diathesis, encephalitis, encephalomyelitis, and, eventually, death of the animal. Acute infections of pregnant females with *Sarcocystis* spp. are associated with fetal death, abortion, and premature birth (Dubey et al. 1988; Dubey and Rommel 1992; Jeffrey 1993). The chronic infections cause economic losses due to reduced quality and quantity of animal meat (Fayer and Elsasser 1991). Apart from domestic ruminants (Tenter 1995), *Sarcocystis* spp. were found in several species of cervids, such as reindeer (Dahlgren and Gjerde 2007, Dahlgren et al. 2007, 2008), red deer (Dahlgren and Gjerde 2010), and roe deer (Gjerde 2012).

Three different *Sarcocystis* species, namely *Sarcocystis gracilis*, *Sarcocystis capreolicanis*, and *Sarcocystis* cf. *hofmanni*, were identified in European roe deer using light microscopy (LM), transmission electron microscopy (TEM), and combination thereof (Erber et al. 1978; Entzeroth 1981, 1982; Enzeroth 1985; Sedlaczek and Wesemeier 1995; Santini et al. 1997; Stolte et al. 1996, 1998). The fourth unnamed *Sarcocystis* spp. that was discovered in roe deer with an aid of TEM (Schramlová and Blažek 1978; Entzeroth 1982; Santini et al. 1997) resembled sarcocysts of *Sarcocystis grueneri* found in reindeer (Gjerde 1985), *Sarcocystis cevicanis* in red deer, and *Sarcocystis wapiti* in wapiti (Hernandez et al. 1981; Speer and Dubey 1982). The last *Sarcocystis* spp. identified in roe deer with an aid of morphological methods (Stolte et al. 1997) has similar morphology as *Sarcocystis ovalis* (Dahlgren and Gjerde 2010).

The accuracy of *Sarcocystis* spp. identification and appropriate characterization of phylogenetic relationships, inferred solely from phenotypic characters, is often uncertain and can be highly subjective. To overcome such problems, the morphological and biological observations were complemented with molecular data obtained with an aid of such techniques as isozyme analysis (O’Donoghue et al. 1986) and, more recently, small subunit ribosomal RNA (*ssu rRNA*) sequencing (Ellis et al. 1995; Fenger et al. 1994; Johnson et al. 1988; Tenter et al. 1992). Species-specific sequences can be detected with the latter method and further amplified by PCR for identification of *Sarcocystis* spp. (Tenter et al. 1994; Stojecki et al. 2012). Amplification of DNA and sequencing of the *ssu rRNA* gene from sarcocyst found in roe deer from Norway confirmed the presence of two *Sarcocystis* spp., the already described *S. gracilis* and *S. capreolicanis*, and revealed the existence of two new species, *Sarcocystis oviformis* (Dahlgren and Gjerde 2009) and *Sarcocystis silva* (Gjerde 2012).

The mitochondrial gene encoding the subunit I of cytochrome oxidase (*cox1*) has proven to be particularly suitable as a molecular marker for taxonomic differentiation and evolutionary studies of different eukaryotic groups. Indeed, it was used recently as a target gene to analyze phylogenetic relationships among 22 *Sarcocystis* spp. of cervids, cattle, and sheep (Gjerde 2013). The analysis of *cox1* sequences allowed a better distinction between the closely related species than the examination of *ssu rRNA* sequences and confirmed the presence of four *Sarcocystis* spp. (*S. gracilis*, *S. capreolicanis*, *S. oviformis*, and *S. silva*) in roe deer.

To date, data on phylogenetic relationships between different *Sarcocystis* spp. found in roe deer were based solely on the analysis of specimens collected in Norway. Therefore, the objectives of the present study included molecular identification of *Sarcocystis* spp. collected from Polish roe deer, their genetic comparison with the same species described by Gjerde et al. in Norway, and analysis of phylogenetic relationships between *Sarcocystis* spp. from Polish and Norwegian roe deer (Dahlgren and Gjerde 2009; Gjerde 2012).

## Material and methods

### Isolation and morphological identification of sarcocysts

The study included 14 sarcocysts isolated from four adult (4- and 5-year-old) roe deer found dead in the fall and winter of 2009 in Greater Poland, Forest District Piaski (animal Rd1 and Rd2) and Forest District Przedborów (animal Rd3 and Rd4). Massive invasion of *Sarcocystis* spp. was diagnosed during necropsy in all animals. Sarcocysts were isolated from skeletal muscles (*musculus latissimus dorsi*), esophagus, heart, and tongue (Table 1). Individual sarcocysts were excised under stereomicroscope, using fine needles. A new needle was used for each specimen. Excised cysts were washed three times in PBS and examined under LM. Subsequently, the material was stored up to 3 months at −20 °C until DNA isolation.

**Table 1:** *Sarcocystis* species isolated from roe deer in Poland and their tissue localization

Isolates no.	Roe deer	Organ/tissue	Species	Accession numbers for *cox1*
1	Rd1	Esophagus	*S. gracilis*	KF898103
2	Rd2	Esophagus	*S. gracilis*	KF898100
3	Rd3	Esophagus	*S. gracilis*	KF898101
4	Rd4	Skeletal muscles	*S. oviformis*	KF898108
5	Rd3	Tongue	*S. oviformis*	KF898109
6	Rd4	Tongue	*S. oviformis*	KF898107
7	Rd4	Heart	*S. gracilis*	KF898104
8	Rd4	Heart	*S. silva*	KF898111
9	Rd1	Skeletal muscles	*S. gracilis*	KF898102
10	Rd2	Skeletal muscles	*S. gracilis*	KF898105
11	Rd3	Skeletal muscles	*S. silva*	KF898112
12	Rd3	Skeletal muscles	*S. silva*	KF898110
13	Rd4	Skeletal muscles	*S. gracilis*	KF898106
14	Rd4	Skeletal muscles	*S. silva*	KF898113

### DNA isolation and amplification of *ssu rRNA* and *cox1* genes

Extraction of genomic and mitochondrial DNA was performed immediately upon thawing, with DNeasy Blood & Tissue Kit (Qiagen GmbH, Germany). Isolation procedure was performed according to the manufacturer’s protocol. Briefly, frozen sarcocysts were thawed on ice, and ATL buffer (180 μl) and proteinase K (20 μl) were added. Samples were mixed by vortexing and incubated at 56 °C, until the cysts were completely lysed. Then, 200 μl of AL buffer and 200 μl of 96 % ethanol were added, and samples were again mixed by vortexing. Subsequently, the DNA-containing mixture was transferred on a column supplied by the manufacturer. The column was washed with AW1 buffer (500 μl) and AW2 buffer (500 μl), and the DNA was eluted with 100 μl of AE buffer.

The *ssu rRNA* gene was amplified by PCR, according to slightly modified procedure described by Dahlgren and Gjerde (2007). PCR was carried with primers shown in Table 2, synthesized at the Genomed SA (Poland). Reaction mix (50 μl) contained template DNA, 10× DreamTaq Green Buffer [KCl, (NH_4_)_2_SO_4_, 20 mM MgCl_2_], 0.2 mM dNTP mix, 0.2 μM of each primer, and 1 U of DreamTaq polymerase (Fermentas). PCR was started with initial denaturation (95 °C, 5 min), followed by 35 cycles of denaturation (95 °C, 30 s), annealing (55 °C, 30 s), elongation (72 °C, 1 kb/min), and final elongation (72 °C, 10 min). The PCR amplification of *cox1* gene was carried out with primers showed in Table 2, synthesized at the BioTeZ GmbH (Germany). Reaction mix (50 μl) contained DNA (1 μl), Phusion HF Buffer (Thermo Scientific), MgCl_2_ at final concentration of 4 mM, 0.5 U of Phusion High-Fidelity DNA Polymerase (Thermo Scientific), 0.2 mM dTNPs mix, and 0.2 μM of each primer. All amplification reactions were started with initial denaturation (98 °C, 30 s), followed by 40 cycles of denaturation (98 °C, 5 s), annealing (52 °C, 30 s), elongation (72 °C, 30 s), and final elongation (72 °C, 10 min). PCR products were analyzed by electrophoresis in 1.5 % agarose gel and stained with ethidium bromide.

**Table 2:** Primers used for amplification and sequencing of *ssu rRNA* and *cox1* genes

Primer	Gene	Sequence	Reference
ERIB1	*ssu rRNA*	5′-ACCTGGTTGATCCTGCCAG-3′	Dahlgren et al. (2007)
Primer 1 L	*ssu rRNA*	5′-CCATGCATGTCTAAGTATAAGC-3′	Dahlgren et al. (2007)
S5	*ssu rRNA*	5′-GTTCGATTCCGGAGAGGGAGC-3′	Dahlgren et al. (2007)
S3	*ssu rRNA*	5′-TTGTTAAAGACGAACTACTGCG-3′	Dahlgren et al. (2007)
Primer B	*ssu rRNA*	5′-GATCCTTCTGCAGGTTCACCTAC-3′	Dahlgren et al. (2007)
S4	*ssu rRNA*	5′-TATCCCCATCACGATGCATAC-3′	Dahlgren et al. (2007)
Primer 3H	*ssu rRNA*	5′-GGCAAATGCTTTCGCAGTAG-3′	Dahlgren et al. (2007)
Primer 4H	*ssu rRNA*	5′-CAGAAACTTGAATGATCTATCG-3′	Dahlgren et al. (2007)
SF1	*cox1*	5′- ATGGCGTACAACAATCATAAAGAA-3′	Gjerde (2013)
SR8D	*cox1*	5′- CATTGCCCATDACTACGCC-3′	Gjerde (2013)
SR5	*cox1*	5′-TAGGTATCATGTAACGCAATATCCAT-3′	Gjerde (2013)
COIRm	*cox1*	5′-CCCAGAGATAATACAAAATGGAA-3′	Gjerde (2013)
GraFor	*cox1*	5′-GGTATCTTTAGTGTTGTTGGTAC-3′	This study
GraRev	*cox1*	5′-CAATGGCTGCCCAGTACTC-3′	This study

*ssu RNA* small subunit ribosomal RNA, *cox1* subunit I of cytochrome oxidase

### Sequencing

The nucleotide sequences of the *ssu rRNA* and *cox1* genes were determined by the BigDye® Terminator v3.1 cycle sequencing (Life Technologies) and analyzed on an ABI fluorescence automated DNA sequencer, at the Genomed SA (Poland) and the LGC Genomics (Germany), respectively. CAP3 software (Huang and Madan 1999) was used to align the nucleotide sequences of *ssu rRNA* and *cox1* genes.

### Sequence alignment, comparison, and phylogenetic analyses

Obtained nucleotide sequences were submitted to BLAST (Altschul et al. 1990), and GenBank database was searched for similar sequences with the use of “blastn” and “megablast” algorithms.

The *ssu rRNA* and *cox1* gene sequences were aligned with ClustalW in default settings (Larkin et al. 2007). MEGA 5.0 (Tamura et al. 2011) was used to find the best nucleotide substitution model and generate maximum-likelihood (ML) phylograms of *ssu rRNA* and *cox1* gene sequences. The Kimura 2-parameter (K2) (Kimura 1980), with gamma distribution and invariable sites (K2 + G + I), was found to be the optimal nucleotide substitution model for construction of the ML tree. Subtree-pruning-regrafting (SPR) algorithm was used in order to construct maximum parsimony (MP) trees for both genes. For *ssu rRNA* gene, phylogenetic reconstruction consisted of 147 sequences, and for *cox1* gene, 327 sequences. Bootstrap method was used to check the reliability of formed phylograms. All sequences used in phylogenetic reconstruction are listed in Table S1.

### Population genetic analysis

*Cox1* gene sequences were analyzed with Arlequin 3.5 Software (Excoffier and Lischer 2010). Genetic diversity values, including haplotype numbers and diversity, substitution numbers, polymorphic sites, and nucleotide diversity, were compared between Polish and Norwegian *Sarcocystis* isolates. Tajima’s *D* and Fu’s *F*_s_ tests were used for the evaluation of genetic structure of the *Sarcocystis* under the population expansion. Population pairwise *F*_st_ estimates were also obtained with Arlequin 3.5 software. Mean distance between groups was calculated with MEGA 5.0 software.

## Results

### Morphological characterization of sarcocysts

**Fig. 1:**
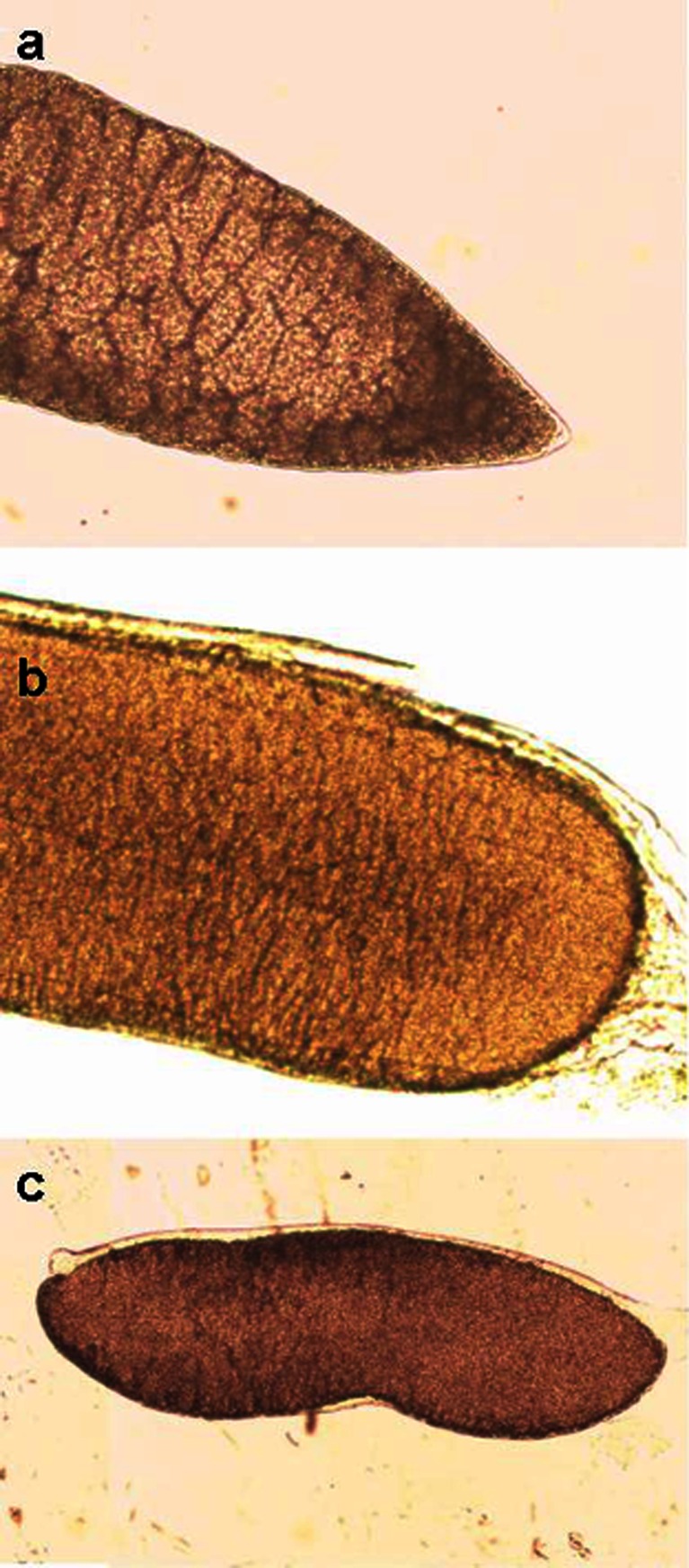
Light microscopic appearance of **a** spindle-shaped fresh cyst (×100), **b** round-ended fresh cyst (×40), and **c** sac-like shaped with fresh cyst with rounded ends (×100)

Several types of sarcocysts were identified in roe deer by light microscopy. Four spindle-shaped sarcocysts (isolates no. 4, 9, 10, and 13; Fig. 1a) and three long cysts with rounded ends (isolates no. 11, 12, and 14; Fig. 1b) were found in skeletal muscles. While the spindle-shaped cysts were similar in size (11.1 to 12.2 mm in length), the long cysts with rounded ends differed significantly in terms of their sizes (7.4 to 10.9 mm in length). All the three cysts isolated from the esophagus were spindle-shaped (isolates no. 1–3) and had similar sizes as those isolated from the muscles (11.1–12.7 mm). In turn, the two *Sarcocystis* residing in the tongue were long cysts with rounded ends (isolates no. 5 and 6) and 7.7 to 9.2 mm in length. The third type of cysts (*n* = 2) with different morphology, described as sac-like with rounded ends (isolates no. 7–8; Fig. 1c), was isolated from the heart. These cysts were markedly smaller (1.5 mm in length) than the other specimens.

### Amplification and sequencing of *ssu rRNA* gene

The samples of genomic DNA from 14 *Sarcocystis* specimens collected from Polish roe deer were used as template to amplify *ssu rRNA* gene by PCR. The sequencing of PCR amplicons revealed the presence of three different nucleotide sequences, designated as ssurRNA1 (1,805 bp), ssurRNA2 (1,833 bp), and ssurRNA3 (1,752 bp). The obtained sequences were subjected to BLAST analysis and then aligned with published sequences of *S. gracilis* (FJ196261, JN226126, JN226127), *S. oviformis* (FJ196262, KC209745, KC209746), and *S. silva* (JN226124, JN226122, JN226123, JN226125 and EU282016) with an aid of ClustalW software (Dahlgren and Gjerde 2009; Gjerde 2012, 2013). The seven sequences (isolates no. 1, 2, 3, 7, 9, 10, and 13) designated as ssurRNA1 turned out to be almost identical (99.9 %) with the sequence of *ssu rRNA* gene of Norwegian *S. gracilis*. Another three sequences (isolates no. 4, 5, and 6) referred to as ssurRNA2 were almost identical (99.9 %) with the sequence of *ssu rRNA* gene of *S. oviformis*, and four ssurRNA3 sequences (isolates no. 8, 11, 12, and 14) closely resembled (99.37–99.66 %, average identity 99.56 %) previously reported sequences of *ssu rRNA* gene from *S. silva* (Table 3). All three newly identified sequences, ssurRNA1 (*ssu rRNA* gene of *S. gracilis*), ssurRNA2 (*ssu rRNA* gene of *S. oviformis*), and ssurRNA3 (*ssu rRNA* gene of *S. silva*), were submitted to the GenBank and given the accession numbers KF880741, KF880742, and KF880743, respectively.

**Table 3:** Intraspecific sequence similarity (%) between *ssu rRNA* genes of *S. silva* isolates from different geographical areas

	SsP (KF880743)	SsN (JN226124)	SsN (JN226122)	SsN (JN226123)	SsN (JN226125)	SsN (EU282016)
SsP (KF880743)	100.00	99.66	99.49	99.37	99.66	99.60
SsN (JN226124)	99.66	100.00	99.83	99.60	99.83	99.83
SsN (JN226122)	99.49	99.83	100.00	99.43	99.66	99.66
SsN (JN226123)	99.37	99.60	99.43	100.00	99.77	99.77
SsN (JN226125)	99.66	99.83	99.66	99.77	100.00	100.00
SsN (EU282016)	99.60	99.83	99.66	99.77	100.00	100.00

*SsP S. silva* of Polish origin, *SsN S. silva* of Norwegian origin

The sequences of Polish *S. gracilis* and *S. oviformis ssu rRNA* genes differed from their Norwegian counterparts only by one nucleotide. The *ssu rRNA* gene from Polish *S. gracilis* isolates had an insertion of T in position 682, and *ssu rRNA* gene from Polish *S. oviformis* had a substitution of T to A in position 187. Five nucleotide differences, unique for Polish sequence, were identified in the case of *ssu rRNA* gene from Polish *S. silva* sarcocysts. A transversion of C for G was found in position 3 when the Polish sequence was compared with Norwegian sequences designated as JN226122, JN226123, JN226124, and EU282016, and another transversion of A for C was identified in position 5 as compared to Norwegian sequences designated as JN226122, JN226123, JN226124, and EU282016. Moreover, an insertion of G in position 885, deletion of C in position 1468, and transition of G for A in position 1736 were found in the Polish sequence when compared to all the Norwegian sequences (Table S2).

**Fig. 2:**
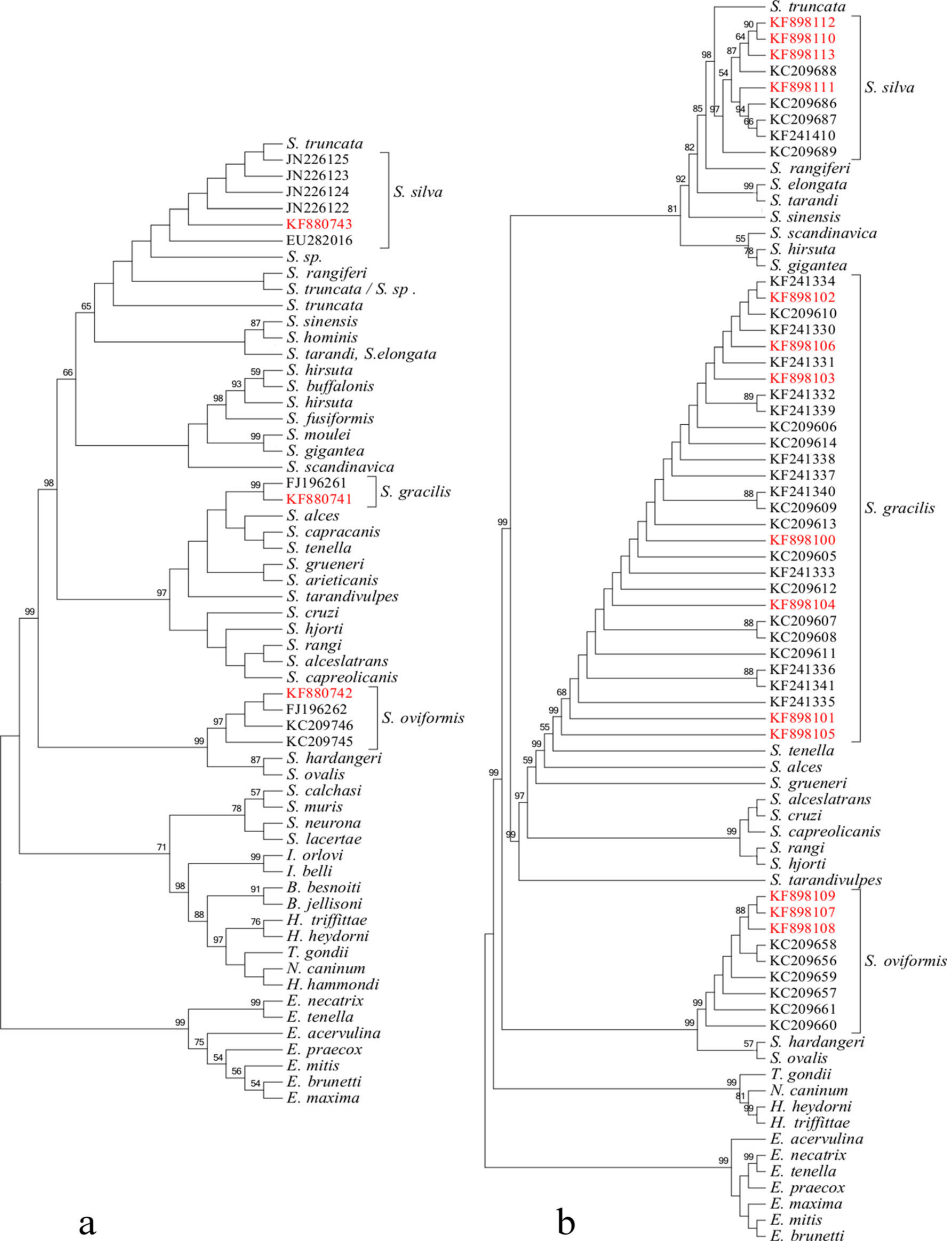
Maximum parsimony of DNA phylograms of selected *Sarcocystidae* and *Coccidia*. **a** The *ssu rRNA* tree was constructed based on the alignment of nearly complete *ssu rRNA* gene sequences of 14 Polish *Sarcocystis* spp. isolates and available *ssu rRNA* gene sequences of related species deposited in GenBank. **b** The *cox1* tree was constructed based on the alignment of nearly complete *ssu rRNA* gene sequences of 14 Polish *Sarcocystis* spp. isolates and available *ssu rRNA* gene sequences of related species deposited in GenBank. Trees were rooted with *Eimeria spp.* The italicized values among the branches indicate percent bootstrap value per 1,000 replicates. Bootstrap values below 50 % are not shown. Polish isolates are marked in red. The GenBank accession numbers of all sequences used for construction of the trees are given in Table S1 (color figure online)

The phylogenetic relationships among Polish and Norwegian *Sarcocystis* isolates were established by constructing phylogenetic trees obtained with the ML and MP methods, as reported previously by Gjerde (2013) for *Sarcocystis* species from cervids, cattle, and sheep. Both methods produced similar results: Only minor differences in branching of aligned sequences were observed, mostly in the case of branches with low bootstrap values. Phylogenetic tree based on *ssu rRNA* gene sequences, obtained by the MP method, is shown in Fig. 2a. Polish isolates of *S. gracilis* represented a sister leaf to Norwegian isolates with high bootstrap value (99 %), dividing these sequences. Polish *S. oviformis* isolates were nested in three Norwegian isolates. All *S. silva* isolates formed clade with one isolate of *Sarcocystis truncata*. Thus, phylogenetic analysis confirmed close relationships between all the Polish and Norwegian isolates of *Sarcocystis* species, namely *S. gracilis*, *S. oviformis*, and *S. silva*.

### Amplification and sequencing of *cox1* gene

Mitochondrial DNA isolated from the abovementioned *Sarcocystis* specimens was used as a template to amplify the sequences of *cox1* gene by PCR. All the obtained amplicons were sequenced, and the sequences were submitted to GenBank and given the accession numbers shown in Table 1. The sequencing of PCR amplicons produced three sets of closely related sequences, designated as cox1.I, cox1.II, and cox1.III.

As for *ssu rRNA* gene, the *cox1* sequences of Polish roe deer isolates were first subjected to BLAST analysis, and then ClustalW software was used to align them with the published sequences of *cox1* gene from Norwegian isolates of *S. gracilis* (KC209605–KC209614, KF241330–KF241341), *S. oviformis* (KC209656–KC209661), and *S. silva* (KC209686–KC209689, KF241410) (Gjerde 2013, 2014). Based on these analyses, cox1.I sequences (isolates no. 1, 2, 3, 7, 9, 10, and 13) were identified as *S. gracilis*, cox1.II sequences (isolates no. 4, 5, and 6) as *S. oviformis*, and cox1.III sequences (isolates no. 8, 11, 12, and 14) as *S. silva*.

**Fig. 3:**
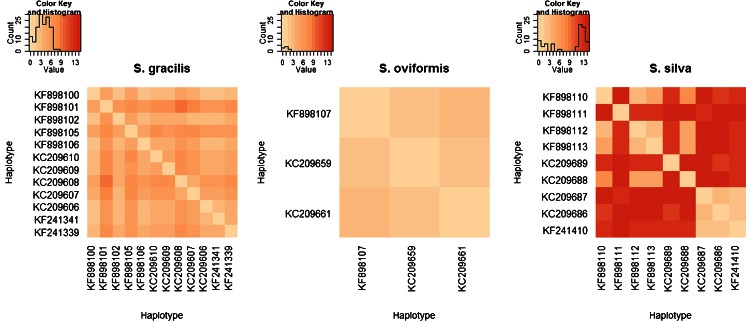
Interhaplotypic distance matrix for *S. gracilis*, *S. oviformis*, and *S. silva* haplotypes. Heat map for each species shows differences between haplotypes. GenBank accession numbers of one representative strain from each haplotype are displayed on the *x-* and *y-axes*. Full list of isolates belonging to presented haplotypes is shown in Tables S2 and S3. A color key containing histogram is added to each chart. “Value” on the *x-axes* and the *color gradient* correspond to the number of nucleotide differences between two haplotypes. “Count” on the *y-axes* describes the number of squares with occurring value

Clustal alignment was further analyzed with Arlequin 3.5 software for the purpose of population genetic analysis. Generally, five haplotypes were found when Polish *S. gracilis* isolates were compared with each other. The comparison of *cox1* gene sequences identified in our study with Norwegian sequences revealed that both groups were characterized by similar haplotype frequency and nucleotide diversity (Table 4). The average number of pairwise differences for Polish isolates was 1.9 (±1.4) with 6 variable sites, and for Norwegian isolates 2.6 (±1.6) with 16 variable sites. Figure 3a presents interhaplotypic distance matrix for all *S. gracilis* isolates. Sequence identity between Polish isolates of *S. gracilis* was 99.33–100 % (average identity 99.70 %), and the identity between Polish and Norwegian amounted to 99.11–100 % (average identity 99.72 %) (Table S3). Between-group mean distance value for *S. gracilis* was 0.0024.

**Table 4:** Summary statistics of population genetic diversity of *cox1* gene sequences from *Sarcocystis* spp.

Species	Origin	N/H	Hd	Trs/Trv	S	Theta-π	π (average ± SD)	Tajima’s *D* (*p* value)	Fu’s *F*_s_ (*p* value)
*S. gracilis*	Poland	7/5	0.8571 ± 0.1371	6/0	6	1.90476 ± 1.40682	0.002869 ± 0.002119	−1.12898 (0.15900)	1.22214 (0.74000)
	Norway	22/10	0.8701 ± 0.0522	13/3	16	2.63203 ± 1.63003	0.002759 ± 0.001709	−1.45418 (0.05200)	−2.93961 (0.05400)
	All	29/12	0.8695 ± 0.0421	17/3	20	2.30788 ± 1.518425	0.002466 ± 0.001562	−1.91793 (0.01200)	5.19798 (0.96400)
*S. oviformis*	Poland	3/1	0.0000 ± 0.0000	0/0	0	0.00000 ± 0.00000	0.000000 ± 0.000000	0.00000 (1.00000)	0.00000 (N.A.)
	Norway	6/2	0.6000 ± 0.1291	0/1	1	0.60000 ± 0.63246	0.000611 ± 0.000644	1.44510 (0.97400)	0.79518 (0.79518)
	All	9/3	0.7500 ± 0.0786	0/2	2	1.00000 ± 0.83887	0.001018 ± 0.000854	1.23476 (0.89700)	0.35118 (0.49400)
*S. silva*	Poland	4/4	1.0000 ± 0.1768	13/2	15	7.66667 ± 5.41242	0.008306 ± 0.005864	−0.64018 (0.35300)	0.01708 (0.27400)
	Norway	5/5	1.0000 ± 0.1265	16/4	20	11.00000 ± 7.59264	0.009967 ± 0.006467	−0.30802 (0.48200)	−0.44982 (0.22700)
	All	9/9	1.0000 ± 0.0524	24/4	27	10.16667 ± 5.82276	0.011015 ± 0.006309	0.11680 (0.58300)	−2.77120 (0.04300)

*N* number of isolates, *H* number of haplotypes, *Hd* haplotype diversity, *Trs* number of transitions, *Trv* number of transversions, *S* number of polymorphic sites, *Theta-π* average number of pairwise differences, *π* nucleotide diversity, *N.A.* not available

Each of the three Polish isolates of *S. oviformis* had the same nucleotide sequence of *cox1* gene. The average number of pairwise differences for Polish isolates was 0 (±0) with no variable sites, and for Norwegian isolates 0.6 (±0.6) with one variable site. Figure 3b presents interhaplotypic distance matrix for all *S. oviformis* isolates. Sequence identity between Polish and Norwegian isolates was 99.85 % (Table S4). Between-group mean distance value for *S. oviformis* was 0.0015.

*S. silva* showed the highest genetic variability. Each of the four Polish and five Norwegian isolates had unique sequences. The average number of pairwise differences for Polish isolates was 7.6 (±5.4) with 15 variable sites, and for Norwegian isolates 11.0 (±7.6) with 20 variable sites. Figure 3c presents interhaplotypic distance matrix for all *S. silva* isolates. The identity of sequences between Polish isolates ranged from 98.48 to 99.78 % (average identity 99.17 %) and between Polish and Norwegian from 98.48 to 99.57 % (average identity 98.76 %) (Table S5). Between-group mean distance value for *S. silva* was 0.016. Tajima’s *D* and Fu’s *F*_s_ tests showed no significant differences between any of the species, (*p* > 0.05). Pairwise *F*_st_ values for Polish and Norwegian populations showed no significant differences (data not shown).

As for *ssu rRNA* gene, phylogenetic analyses based on *cox1* gene gave similar results for the MP and ML methods. Minor differences in branching of aligned sequences were found in branches with low bootstrap values. The analysis of phylogenetic tree based on MP (Fig. 2b) revealed that the Polish sequences of *cox1* gene of all the three identified *Sarcocystis* species co-localized with other *cox1* sequences representing the same species. The Polish isolates of *S. gracilis* were distributed among the Norwegian isolates and did not form a separate group. Similarly, Polish *S. oviformis* isolates were nested in a clade of all Norwegian isolates. In turn, *S. silva* sequences were divided in three clades. The first of them consisted of three Polish isolates and one Norwegian isolate. The second clade consisted of one Polish and three Norwegian isolates. The first and the second clades turned out to be sister clades. The third clade comprised one Norwegian sequence and represented a sister clade for the remaining two clades.

## Discussion

The molecular studies on *Sarcocystis* species, which parasatize cervids, are rare, (Gjerde 2013) and such studies were not performed so far in Poland. Therefore, the present study was undertaken to identify the *Sarcocystis* spp. parasitizing Polish roe deer and to compare their characteristics with those of Norwegian isolates, as available data on the specimens collected in various geographical locations are sparse.

Polish sarcocysts isolated from various tissues differed in terms of their shape and size. *S. gracilis* and *S. silva* found in the skeletal muscles, esophagus, and tongue had morphology typical for the species but were larger than the respective Norwegian isolates (Dahlgren and Gjerde 2009; Gjerde 2012). In contrast, the myocardial cysts of both species were generally smaller and sac-like shaped. Generally, the morphology of cysts found in Polish roe deer was similar to that of Norwegian isolates representing the same *Sarcocystis* species (Dahlgren and Gjerde 2009; Gjerde 2012). However, Polish and Norwegian isolates turned out to differ considerably in terms of their size distribution. The length of *S. gracilis* cysts isolated from the skeletal muscles, esophagus, and myocardium of Polish roe deer was 6.7, 12.2, and 1.5 mm, respectively. In turn, the size of typical *S. gracilis* cysts from Norwegian roe deer ranges from 1.0 to 2.5 mm for diaphragm isolates and between 0.3 and 0.9 mm for myocardial isolates (Dahlgren and Gjerde 2009). Cysts of Polish *S. silva* found in skeletal muscles and heart measured 7.4–11.5 and 1.5 mm, respectively. Similar to other Polish species, the cysts were larger than those isolated and described by Gjerde (2012) in Norway (0.6 and 0.55 mm, respectively). It should be noted, however, that the Norwegian cysts were isolated from the esophagus. The Polish cysts of *S. oviformis* isolated from the tongue and skeletal muscles measured about 7.7–9.2 and 10.9 mm, respectively. Cysts of the same species described by Dahlgren and Gjerde (2009) were much smaller (1.6–2.0 mm), but again they were isolated from other tissues, namely the esophagus and diaphragm. Also, the study by Pérez-Creo et al. (2013), who analyzed the prevalence and morphology of *Sarcocystis* spp. in Spanish roe deer, has shown that cysts of different species isolated from the diaphragm and myocardium were smaller than 1.5 mm. Larger cysts (mean size of 2.055 mm) were observed solely in the esophagus. The abovementioned discrepancies in the size of Polish, Norwegian, and Spanish *Sarcocystis* isolates may result from their different localization. The Polish sarcocysts were isolated from *musculus latissimus dorsi*, whereas the Norwegian and Spanish isolates originated from the diaphragm (Dahlgren and Gjerde 2009; Gjerde 2012; Pérez-Creo et al. 2013). Moreover, it should be remembered that only the largest specimens were included in our study.

The hereby described morphological characteristics of cysts isolated from Polish roe deer and their comparison with the respective data of Norwegian specimens confirm that the identification of *Sarcocystis* spp. solely on the basis of phenotypic characterization is inaccurate. In order to overcome this issue, the results of morphological analyses should be supported by DNA-based molecular techniques based on sequencing of *ssu rRNA* and *cox1* genes (Gjerde 2013).

The analysis of *ssu rRNA* and *cox1* genes from sarcocysts isolated from Polish roe deer revealed the presence of the same three species: *S. gracilis*, *S. silva*, and *S. oviformis*, as in Norwegian roe deer (Dahlgren and Gjerde 2009; Gjerde 2012). However, we were unable to isolate the fourth *Sarcocystis* species found in roe deer, *S. capreolicanis* (Gjerde 2012), probably due to the limited number of available samples. The sequence analysis of *ssu rRNA* gene revealed the lack of differences between *Sarcocystis* isolates belonging to one species, and a very low degree of genetic diversity, close to zero, between Polish and Norwegian sarcocysts (the intraspecific variation rate was 99.9 % for *S. gracilis* and *S. oviformis* and 99.56 % for *S. silva*). Thus, the *Sarcocystis* specimens representing three species isolated from roe deer living in different geographical areas displayed a very low level of genetic diversity.

Contrary to the results of *ssu rRNA* analysis, small intraspecies differences in *cox1* sequences were found among the Polish *Sarcocystis* isolates. Five haplotypes were found when *S. gracilis* isolates were compared to each other, with average identity of 99.70 %. Similarly, four haplotypes were identified on comparison of *S. silva* isolates, which corresponded to a 99.17 % identity on average. In turn, only one haplotype was found in the case of *S. ovifiormis*.

The comparison of Polish and Norwegian isolates representing the same *Sarcocystis* species revealed similar degree of sequence identity, namely 99.72 % for *S. gracilis*, 98.76 % for *S. silva*, and 99.85 % for *S. oviformis*.

High degree of identity between Polish and Norwegian isolates was further confirmed by the results of genetic population analysis; the values of haplotype diversity (*H*_d_), average number of pairwise differences (theta-π), and nucleotide diversity (π) turned out to be similar for sequences obtained in Norway and Poland. On phylogenetic reconstruction, Polish isolates were not separated from their Norwegian counterparts. This conclusion was further supported by spatially nonrestricted display of haplotypes on heat maps (Fig. 3).

Gjerde (2013, 2014) put into question the usefulness of *ssu rRNA* gene in phylogenetic studies of *Sarcocystis*; this author found that discriminatory power of analyses based on sequences of *cox1* gene is higher than the analyses based on the sequencing of *ssu rRNA* gene. This observation is consistent with data published by Ogedengbe et al. (2011), who showed that partial *cox1* sequences are more useful for identifying *Eimeria* species than nearly complete *ssu rRNA* gene sequences. Moreover, *cox1* gene sequences turned out to be more accurate when used to reveal phylogenetic relationships among closely related taxa. This is confirmed by our findings presented in Table 5, illustrating pairwise distances between species for *S. gracilis*, *S. oviformis*, and *S. silva* determined on the basis of *ssu rRNA* and *cox1* sequences. The genetic matrix based on *cox1* sequences points to a higher resolution of interspecies genetic differences than in the case of *ssu rRNA* sequences. Similar results were obtained in the case of other species subjected to phylogenetic analyses (data not shown).

**Table 5:** Comparison of mean pairwise differences of *ssu rRNA* sequences (lower block) and *cox1* sequences (upper block) between *S.gracilis*, *S.oviformis* and *S.silva* species

	*S. gracilis*	*S. oviformis*	*S. silva*
*S. gracilis*		0.885	0.488
*S. oviformis*	0.098		0.795
*S. silva*	0.053	0.085	

To summarize, our study revealed that the same *Sarcocystis* species isolated from the same hosts living in different geographic regions show a considerable level of genetic similarity. These results are consistent with previously published findings by Gjerde (2014), who observed that *Sarcocystis* spp. from cervids, namely *S. elongata and S. truncata*, show a low degree of intraspecies variation in the *ssu rRNA* gene. Furthermore, the other species (e.g., *S. gracilis, S. oviformis*) found in cervids showed little or no variation in this gene, which suggests that (nearly) identical sequences can be obtained from different sarcocysts isolated from different animals. The limited genetic diversity of *Sarcocystis* isolates belonging to the same species was also observed in the case of *Sarcocystis neurona*; in the case of this species, analysis of multiple genetic markers turned out to be insufficient to distinguish between terrestrial isolates and strains infecting marine mammals (Wendte et al. 2010). The results of a study of closely related species *Toxoplasma gondii* (Grigg and Sundar 2009) suggest that the phenomenon of limited genetic diversity among *Sarcocystis* isolates can result from the ability of few haplotypes to clonal propagation. However, further research, including more isolates from various countries and continents, is required in order to confirm this hypothesis.

## Electronic supplementary material

Below is the link to the electronic supplementary material.

Table S1 GenBank accession numbers of *ssu rRNA* and *cox1* gene sequences used for phylogenetic reconstructions. (DOCX 12 kb)

Table S2 Nucleotide variation in *ssu rRNA* gene sequences of Polish (KF880743) and Norwegian (JN226122-JN226125, EU282016) *S. silva* isolates. Nucleotides identical to the nucleotide sequence of KF880743 are marked by dots. Dashes indicate sites with no nucleotide aligned. The site numbers on the top of each gene sequence correspond to position of nucleotides in KF880743 sequence. (DOCX 13 kb)

Table S3 Intraspecific similarity (%) between partial *cox1* gene sequences of *S. gracilis* isolates from different geographical areas. Isolates belonging to given haplotypes: F898100= KF898103= KF898104= KC209614= KC209613= KF241338= KF241337; KF898102= KF241333, KF898106= KC209612= KC209611= KC209605= KF241335= KF241334= KF241331= KF241330; KC209609= KF241340; KF241341= KF241336; KF241339= KF241332 (DOCX 14 kb)

Table S4 Intraspecific similarity (%) between partial *cox1* gene sequences of *S. oviformis* isolates from different geographical areas. List of isolates belonging to a given haplotype: KF898107 = KF898108 = KF898109; KC209659 = KC209656 = KC209658; KC209661 = KC209657 = KC209660. (DOCX 12 kb)

Table S5 Intraspecific similarity (%) between partial *cox1* gene sequences of *S. silva* isolates from different geographical areas. (DOCX 13 kb)
