# Supplementary material for: Molecular characterization of Sarcocystis species from Polish roe deer based on ssu rRNA and cox1 sequence analysis
Source: Parasitol Res. 2014 Jun 20;113(8):3029–39. doi: 10.1007/s00436-014-3966-x (PMC4110405; doi:10.1007/s00436-014-3966-x)
Supplement: Supplementary file 1 — GenBank accession numbers of ssu rRNA and cox1 gene sequences used for phylogenetic reconstructions. (DOCX 12 kb) [file 436_2014_3966_MOESM1_ESM.docx]

**Molecular characterization of *Sarcocystis* species from Polish roe deer based on**

***ssu rRNA* and *cox1* sequence analysis**

**Rafał Kolenda^1^ , Maciej Ugorski^2, 3^ , Michał Bednarski^4,^***

Brandenburg University of Technology Cottbus– Senftenberg, Faculty of Natural Sciences, Großenhainer Str. 57, D-01968, Senftenberg, Germany^1^

Department of Biochemistry, Pharmacology and Toxicology^2^ , Department of Epizootiology and Clinic of Bird and Exotic Animals^4^ , Wrocław University of Environmental and Life Sciences, 50-375 Wrocław, Poland

Laboratory of Glycobiology and Cell Interactions, Ludwik Hirszfeld Institute of Immunology and Experimental Therapy, Polish Academy of Sciences, 53-114 Wrocław, Poland^3^

*** Corresponding author:**

Michał Bednarski; Mailing addres : Department of Epizootiology and Clinic of Bird and Exotic Animals , Wrocław University of Environmental and Life Sciences, 50-375 Wrocław, Poland; Fax: +48 713205336; E-mail: [michal.bednarski@up.wroc.pl](mailto:michal.bednarski@up.wroc.pl)

**Table S1.** GenBank accession numbers of *ssu rRNA* and *cox1* gene sequences used for phylogenetic reconstructions.

| *ssu rRNA* | *cox1* |
| --- | --- |
| AB251926, AB257085, AB257086, AB682779, AB682780, AB257154- AB257162, GQ251011- GQ251030, GQ250967- GQ250990, GQ984222, GQ984224, GQ245670, EF056010- EF056012, EF056014- EF056018, EF472967, EF467654- EF467657, JX679466, JX679467, JX679469- JX679471, KC209733- KC209746, EU282016- EU282034, JN226117- JN226119, JN226122- JN226125, AY365026, AY015113, DQ060683, DQ538348, AF017122, AF017121, AF109678, AF291426, AF096498, U16159, U40262, U67115, U67116, U67119- U67121, U03071, U07812, L76472, L76473, L24382, L24384, M64244, KF880741- KF880743, FJ196261, FJ196262, | KC209578-KC209732, KF898100- KF898113, KF241309- KF241452, JN864949, HQ702479- HQ702484, JX473247- JX473253 |
