# Supplementary material for: Molecular characterization of Sarcocystis species from Polish roe deer based on ssu rRNA and cox1 sequence analysis
Source: Parasitol Res. 2014 Jun 20;113(8):3029–39. doi: 10.1007/s00436-014-3966-x (PMC4110405; doi:10.1007/s00436-014-3966-x)
Supplement: Supplementary file 2 — Nucleotide variation in ssu rRNA gene sequences of Polish (KF880743) and Norwegian (JN226122-JN226125, EU282016) S. silva isolates. Nucleotides identical to the nucleotide sequence of KF880743 are marked by dots. Dashes indicate sites with no nucleotide aligned. The site numbers on the top of each gene sequence correspond to position of nucleotides in KF880743 sequence. (DOCX 13 kb) [file 436_2014_3966_MOESM2_ESM.docx]

**Molecular characterization of *Sarcocystis* species from Polish roe deer based on**

***ssu rRNA* and *cox1* sequence analysis**

**Rafał Kolenda^1^ , Maciej Ugorski^2, 3^ , Michał Bednarski^4,^***

Brandenburg University of Technology Cottbus– Senftenberg, Faculty of Natural Sciences, Großenhainer Str. 57, D-01968, Senftenberg, Germany^1^

Department of Biochemistry, Pharmacology and Toxicology^2^ , Department of Epizootiology and Clinic of Bird and Exotic Animals^4^ , Wrocław University of Environmental and Life Sciences, 50-375 Wrocław, Poland

Laboratory of Glycobiology and Cell Interactions, Ludwik Hirszfeld Institute of Immunology and Experimental Therapy, Polish Academy of Sciences, 53-114 Wrocław, Poland^3^

*** Corresponding author:**

Michał Bednarski; Mailing addres : Department of Epizootiology and Clinic of Bird and Exotic Animals , Wrocław University of Environmental and Life Sciences, 50-375 Wrocław, Poland; Fax: +48 713205336; E-mail: [michal.bednarski@up.wroc.pl](mailto:michal.bednarski@up.wroc.pl)

**Table S2**. Nucleotide variation in *ssu rRNA* gene sequences of Polish (KF880743) and Norwegian (JN226122-JN226125, EU282016) *S. silva* isolates. Nucleotides identical to the nucleotide sequence of KF880743 are marked by dots**.** Dashes indicate sites with no nucleotide aligned. The site numbers on the top of each gene sequence correspond to position of nucleotides in KF880743 sequence.

|  | Nucleotide position | 3 | 5 | 9  8 | 5  9  5 | 6  4  2 | 6  6  5 | 7  2  5 | 7  2  9 | 7  4  2 | 7  8  6 | 7  8  7 | 7  8  8 | 8  8  5 | 1  4  6  8 | 1  7  3  6 |
| --- | --- | --- | --- | --- | --- | --- | --- | --- | --- | --- | --- | --- | --- | --- | --- | --- |
| *S.silva* | KF880743 | G | C | C | T | C | T | T | T | G | - | - | - | G | - | A |
|  | JN226124 | C | A | . | . | . | . | A | . | . | - | - | - | - | C | G |
|  | JN226122 | C | A | T | . | T | C | A | . | . | - | - | - | - | C | G |
|  | JN226123 | C | A | . | C | . | . | . | C | A | T | T | G | - | C | G |
|  | JN226125 |  |  | . | . | . | . | . | C | A | - | - | - | - | C | G |
|  | EU282016 | C | A | . | . | . | . | . | C | A | - | - | - | - | C | G |
