# Supplementary material for: Molecular characterization of Sarcocystis species from Polish roe deer based on ssu rRNA and cox1 sequence analysis
Source: Parasitol Res. 2014 Jun 20;113(8):3029–39. doi: 10.1007/s00436-014-3966-x (PMC4110405; doi:10.1007/s00436-014-3966-x)
Supplement: Supplementary file 3 — Intraspecific similarity (%) between partial cox1 gene sequences of S. gracilis isolates from different geographical areas. Isolates belonging to given haplotypes: F898100= KF898103= KF898104= KC209614= KC209613= KF241338= KF241337; KF898102= KF241333, KF898106= KC209612= KC209611= KC209605= KF241335= KF241334= KF241331= KF241330; KC209609= KF241340; KF241341= KF241336; KF241339= KF241332 (DOCX 14 kb) [file 436_2014_3966_MOESM3_ESM.docx]

**Molecular characterization of *Sarcocystis* species from Polish roe deer based on**

***ssu rRNA* and *cox1* sequence analysis**

**Rafał Kolenda^1^ , Maciej Ugorski^2, 3^ , Michał Bednarski^4,^***

Brandenburg University of Technology Cottbus– Senftenberg, Faculty of Natural Sciences, Großenhainer Str. 57, D-01968, Senftenberg, Germany^1^

Department of Biochemistry, Pharmacology and Toxicology^2^ , Department of Epizootiology and Clinic of Bird and Exotic Animals^4^ , Wrocław University of Environmental and Life Sciences, 50-375 Wrocław, Poland

Laboratory of Glycobiology and Cell Interactions, Ludwik Hirszfeld Institute of Immunology and Experimental Therapy, Polish Academy of Sciences, 53-114 Wrocław, Poland^3^

*** Corresponding author:**

Michał Bednarski; Mailing addres : Department of Epizootiology and Clinic of Bird and Exotic Animals , Wrocław University of Environmental and Life Sciences, 50-375 Wrocław, Poland; Fax: +48 713205336; E-mail: [michal.bednarski@up.wroc.pl](mailto:michal.bednarski@up.wroc.pl)

**Table S3.** Intraspecific similarity (%) between partial *cox1* gene sequences of *S. gracilis* isolates from different geographical areas.

|  | KF898100 | KF898101 | KF898102 | KF898105 | KF898106 | KC209610 | KC209609 | KC209608 | KC209607 | KC209606 | KF241341 | KF241339 |
| --- | --- | --- | --- | --- | --- | --- | --- | --- | --- | --- | --- | --- |
| KF898100 | 100,00 | 99,56 | 99,89 | 99,56 | 99,89 | 99,78 | 99,78 | 99,56 | 99,67 | 99,89 | 99,89 | 99,78 |
| KF898101 | 99,56 | 100,00 | 99,44 | 99,33 | 99,44 | 99,33 | 99,33 | 99,11 | 99,22 | 99,44 | 99,44 | 99,33 |
| KF898102 | 99,89 | 99,44 | 100,00 | 99,56 | 99,78 | 99,67 | 99,67 | 99,67 | 99,78 | 99,78 | 99,78 | 99,67 |
| KF898105 | 99,56 | 99,33 | 99,56 | 100,00 | 99,56 | 99,33 | 99,44 | 99,33 | 99,33 | 99,44 | 99,44 | 99,44 |
| KF898106 | 99,89 | 99,44 | 99,78 | 99,56 | 100,00 | 99,67 | 99,67 | 99,44 | 99,56 | 99,78 | 99,78 | 99,67 |
| KC209610 | 99,78 | 99,33 | 99,67 | 99,33 | 99,67 | 100,00 | 99,56 | 99,33 | 99,44 | 99,67 | 99,67 | 99,56 |
| KC209609 | 99,78 | 99,33 | 99,67 | 99,44 | 99,67 | 99,56 | 100,00 | 99,33 | 99,44 | 99,67 | 99,67 | 99,56 |
| KC209608 | 99,56 | 99,11 | 99,67 | 99,33 | 99,44 | 99,33 | 99,33 | 100,00 | 99,67 | 99,44 | 99,44 | 99,33 |
| KC209607 | 99,67 | 99,22 | 99,78 | 99,33 | 99,56 | 99,44 | 99,44 | 99,67 | 100,00 | 99,56 | 99,56 | 99,44 |
| KC209606 | 99,89 | 99,44 | 99,78 | 99,44 | 99,78 | 99,67 | 99,67 | 99,44 | 99,56 | 100,00 | 99,78 | 99,67 |
| KF241341 | 99,89 | 99,44 | 99,78 | 99,44 | 99,78 | 99,67 | 99,67 | 99,44 | 99,56 | 99,78 | 100,00 | 99,67 |
| KF241339 | 99,78 | 99,33 | 99,67 | 99,44 | 99,67 | 99,56 | 99,56 | 99,33 | 99,44 | 99,67 | 99,67 | 100,00 |

Isolates belonging to given haplotypes: F898100= KF898103= KF898104= KC209614= KC209613= KF241338= KF241337; KF898102= KF241333, KF898106= KC209612= KC209611= KC209605= KF241335= KF241334= KF241331= KF241330; KC209609= KF241340; KF241341= KF241336; KF241339= KF241332
