# Supplementary material for: Molecular characterization of Sarcocystis species from Polish roe deer based on ssu rRNA and cox1 sequence analysis
Source: Parasitol Res. 2014 Jun 20;113(8):3029–39. doi: 10.1007/s00436-014-3966-x (PMC4110405; doi:10.1007/s00436-014-3966-x)
Supplement: Supplementary file 4 — Intraspecific similarity (%) between partial cox1 gene sequences of S. oviformis isolates from different geographical areas. List of isolates belonging to a given haplotype: KF898107 = KF898108 = KF898109; KC209659 = KC209656 = KC209658; KC209661 = KC209657 = KC209660. (DOCX 12 kb) [file 436_2014_3966_MOESM4_ESM.docx]

**Molecular characterization of *Sarcocystis* species from Polish roe deer based on**

***ssu rRNA* and *cox1* sequence analysis**

**Rafał Kolenda^1^ , Maciej Ugorski^2, 3^ , Michał Bednarski^4,^***

Brandenburg University of Technology Cottbus– Senftenberg, Faculty of Natural Sciences, Großenhainer Str. 57, D-01968, Senftenberg, Germany^1^

Department of Biochemistry, Pharmacology and Toxicology^2^ , Department of Epizootiology and Clinic of Bird and Exotic Animals^4^ , Wrocław University of Environmental and Life Sciences, 50-375 Wrocław, Poland

Laboratory of Glycobiology and Cell Interactions, Ludwik Hirszfeld Institute of Immunology and Experimental Therapy, Polish Academy of Sciences, 53-114 Wrocław, Poland^3^

*** Corresponding author:**

Michał Bednarski; Mailing addres : Department of Epizootiology and Clinic of Bird and Exotic Animals , Wrocław University of Environmental and Life Sciences, 50-375 Wrocław, Poland; Fax: +48 713205336; E-mail: [michal.bednarski@up.wroc.pl](mailto:michal.bednarski@up.wroc.pl)

**Table S4**. Intraspecific similarity (%) between partial *cox1* gene sequences of *S. oviformis* isolates from different geographical areas.

|  | KF898107 | KC209659 | KC209661 |
| --- | --- | --- | --- |
| KF898107 | 100,00 | 99,90 | 99,80 |
| KC209659 | 99,90 | 100,00 | 99,90 |
| KC209661 | 99,80 | 99,90 | 100,00 |

List of isolates belonging to a given haplotype: KF898107 = KF898108 = KF898109; KC209659 = KC209656 = KC209658; KC209661 = KC209657 = KC209660**.**
