# Supplementary material for: Molecular characterization of Sarcocystis species from Polish roe deer based on ssu rRNA and cox1 sequence analysis
Source: Parasitol Res. 2014 Jun 20;113(8):3029–39. doi: 10.1007/s00436-014-3966-x (PMC4110405; doi:10.1007/s00436-014-3966-x)
Supplement: Supplementary file 5 — Intraspecific similarity (%) between partial cox1 gene sequences of S. silva isolates from different geographical areas. (DOCX 13 kb) [file 436_2014_3966_MOESM5_ESM.docx]

**Molecular characterization of *Sarcocystis* species from Polish roe deer based on**

***ssu rRNA* and *cox1* sequence analysis**

**Rafał Kolenda^1^ , Maciej Ugorski^2, 3^ , Michał Bednarski^4,^***

Brandenburg University of Technology Cottbus– Senftenberg, Faculty of Natural Sciences, Großenhainer Str. 57, D-01968, Senftenberg, Germany^1^

Department of Biochemistry, Pharmacology and Toxicology^2^ , Department of Epizootiology and Clinic of Bird and Exotic Animals^4^ , Wrocław University of Environmental and Life Sciences, 50-375 Wrocław, Poland

Laboratory of Glycobiology and Cell Interactions, Ludwik Hirszfeld Institute of Immunology and Experimental Therapy, Polish Academy of Sciences, 53-114 Wrocław, Poland^3^

*** Corresponding author:**

Michał Bednarski; Mailing addres : Department of Epizootiology and Clinic of Bird and Exotic Animals , Wrocław University of Environmental and Life Sciences, 50-375 Wrocław, Poland; Fax: +48 713205336; E-mail: [michal.bednarski@up.wroc.pl](mailto:michal.bednarski@up.wroc.pl)

**Table S5**. Intraspecific similarity (%) between partial *cox1* gene sequences of *S. silva* isolates from different geographical areas.

|  | KF898110 | KF898111 | KF898112 | KF898113 | KC209689 | KC209688 | KC209687 | KC209686 | KF241410 |
| --- | --- | --- | --- | --- | --- | --- | --- | --- | --- |
| KF898110 | 100,00 | 98,48 | 99,78 | 99,57 | 98,70 | 99,35 | 98,59 | 98,81 | 98,70 |
| KF898111 | 98,48 | 100,00 | 98,70 | 98,70 | 98,59 | 98,48 | 98,70 | 98,48 | 98,59 |
| KF898112 | 99,78 | 98,70 | 100,00 | 99,78 | 98,70 | 99,57 | 98,59 | 98,59 | 98,70 |
| KF898113 | 99,57 | 98,70 | 99,78 | 100,00 | 98,70 | 99,57 | 98,59 | 98,59 | 98,70 |
| KC209689 | 98,70 | 98,59 | 98,70 | 98,70 | 100,00 | 98,48 | 98,59 | 98,81 | 98,70 |
| KC209688 | 99,35 | 98,48 | 99,57 | 99,57 | 98,48 | 100,00 | 98,59 | 98,59 | 98,70 |
| KC209687 | 98,59 | 98,70 | 98,59 | 98,59 | 98,59 | 98,59 | 100,00 | 99,78 | 99,89 |
| KC209686 | 98,81 | 98,48 | 98,59 | 98,59 | 98,81 | 98,59 | 99,78 | 100,00 | 99,89 |
| KF241410 | 98,70 | 98,59 | 98,70 | 98,70 | 98,70 | 98,70 | 99,89 | 99,89 | 100,00 |
